# A Human XPC Protein Interactome—A Resource

**DOI:** 10.3390/ijms15010141

**Published:** 2013-12-23

**Authors:** Abigail Lubin, Ling Zhang, Hua Chen, Victoria M. White, Feng Gong

**Affiliations:** Department of Biochemistry and Molecular Biology, University of Miami Miller School of Medicine, Miami, FL 33156, USA; E-Mails: a.lubin@med.miami.edu (A.L.); lzhang@med.miami.edu (L.Z.); HChen2@med.miami.edu (H.C.); v.white1@umiami.edu (V.M.W.)

**Keywords:** XPC, nucleotide excision repair, Xeroderma pigmentosum, yeast two hybrid

## Abstract

Global genome nucleotide excision repair (GG-NER) is responsible for identifying and removing bulky adducts from non-transcribed DNA that result from damaging agents such as UV radiation and cisplatin. Xeroderma pigmentosum complementation group C (XPC) is one of the essential damage recognition proteins of the GG-NER pathway and its dysfunction results in xeroderma pigmentosum (XP), a disorder involving photosensitivity and a predisposition to cancer. To better understand the identification of DNA damage by XPC in the context of chromatin and the role of XPC in the pathogenesis of XP, we characterized the interactome of XPC using a high throughput yeast two-hybrid screening. Our screening showed 49 novel interactors of XPC involved in DNA repair and replication, proteolysis and post-translational modifications, transcription regulation, signal transduction, and metabolism. Importantly, we validated the XPC-OTUD4 interaction by co-IP and provided evidence that *OTUD4* knockdown in human cells indeed affects the levels of ubiquitinated XPC, supporting a hypothesis that the OTUD4 deubiquitinase is involved in XPC recycling by cleaving the ubiquitin moiety. This high-throughput characterization of the XPC interactome provides a resource for future exploration and suggests that XPC may have many uncharacterized cellular functions.

## 1. Introduction

XPC is a 940 amino acid protein which harbors domains that can bind to damaged DNA and repair factors. In complex with RAD23B and CETN2 [1], XPC recognizes DNA damage based on bulky disfigurations of DNA and recruits TFIIH to these sites, initiating the global genome nucleotide excision repair (GG-NER) pathway. XPC appears to not only recognize various structurally unrelated lesions, but also bind to undamaged DNA with substantial affinity. GG-NER is responsible for identifying and removing bulky adducts from the DNA typically caused by UV radiation. XPC, along with DDB2, has been established as a DNA damage recognition protein for GG-NER.

XPC is one of the seven complementation groups of xeroderma pigmentosum (XP). First described in 1933 with its seven complementation groups and their frequencies characterized in 1991 [2], the molecular mechanism of xeroderma pigmentosum has become better understood though its genotype-phenotype relationship remains complex. Manifesting in children younger than one year old, XP symptoms include photosensitivity, neurological defects, and a predisposition to skin cancers. Patients in *XPC*, the complementation group corresponding to mutations in the gene coding for the XPC protein, can exhibit XP or XP with neurological abnormalities. These patients have been observed with diminished DNA repair capacity, abnormal skin lesions, freckling, atrophy, telangiectasia, hypopigmentation, actinic keratosis, and multiple skin cancers including squamous cell carcinomas, basal cell carcinomas, and melanomas [3], and unusual cases have been reported with neurological symptoms such as those appearing in systemic lupus erythematosus [4], hyperactivity potentially linked to low levels of glycine, and autistic features [5] and ophthalmological symptoms such as the clouding of the cornea, prominent vascular growth on the conjunctiva, and loss of lashes.

XPC is covalently bonded to different modifiers, including ubiquitin and its relative SUMO, during the course of its role in GG-NER [6]. XPC ubiquitination is carried out by the DDB2-associated DDB1-CUL4-ROC1 complex, though the complete role of this modification has yet to be determined. Typically, ubiquitination is indicative of degradation by the ubiquitin-proteasome system; interestingly, the ubiquitination of XPC does not lead to its degradation, indicating an alternative role for XPC ubiquitination. Sugasawa *et al.* indicated that ubiquitination of XPC by the DDB1-CUL4-ROC1 complex increased the affinity of XPC to damaged DNA and is potentially involved in the handoff of 6-4PP repair from DDB2 to XPC. The sumoylation of XPC has been proposed to protect XPC from degradation after UV irradiation. Recently, it has been indicated that XPC is ubiquitinated after sumoylation by RNF111, which serves to promote NER [7]. How XPC is deubiquitinated and removed from sites of damage remains unexplored.

XPC functionally interacts with RAD23B, CETN2, TFIIH, and XPA in the context of NER. XPC functions in GG-NER within the XPC-RAD23B-CETN2 complex; the interaction of XPC and RAD23B has been shown to increase the affinity of XPC for damaged DNA [8] while the interaction between XPC and CETN2 has been shown to stabilize XPC and promote NER [1,9]. The interactions of XPC with CETN2, RAD23B, and XPA have been biochemically characterized [10,11]. Interestingly, XPC can functionally interact with both RAD23B and RAD23A, a homolog of RAD23B [12,13]. XPC interacts with TFIIH to recruit the transcription factor to damaged DNA for the completion of NER [14,15]. XPC-RAD23B can also interact with XPA-RPA [16] and HMG1 [17] to recognize psoralen interstrand crosslinks (ICLs). Some of these interactors are chromatin remodeling factors. For example, XPC has been shown to interact with hSNF5, a component of the SWI/SNF ATP-dependent chromatin remodeling complex, in response to UV radiation [18] and potentially interacts weakly with p150, a subunit of chromatin assembly factor 1 (CAF-1), though this interaction has yet to be confirmed [19]. In base excision repair (BER), XPC interacts with thymine DNA glycosylase (TDG), an initiator of BER which responds to G/T mismatches formed from the deamination of 5-methylcytosines. XPC-RAD23B was shown to form a complex with TDG-bound DNA and stimulate TDG activity [20]. XPC has also been shown to play roles outside damage repair. The XPC-RAD23B-CETN2 complex, shown to interact directly with Oct4 and Sox2, is requisite for stem cell self-renewal and efficient somatic cell reprogramming [21]. Additionally, XPC has been identified in large screenings as interacting with other proteins in as of yet unknown capacities. These proteins include CHRAC1, MECP2, TOP2B, USP11, WRAP53, ZCCHC6 [22], LSM3 [23], SMAD1, ZNF512B [24], and BANF1 [25].

Though the majority of the XP symptoms can be explained by XPC’s role in the GG-NER pathway as a sensor of DNA damage, the causes of some symptoms, particularly those with neurological or ophthalmological effects, are unknown. Discovering the proteins that interact with XPC within the cell and, therefore, the cellular functions of XPC in addition to its role in GG-NER, could provide the understanding necessary to comprehend the full effects of xeroderma pigmentosum. In this study, we used a high-throughput Yeast Two Hybrid screening to elucidate the interactome of XPC. We identified 49 proteins that interact with XPC with roles in DNA repair and replication, proteolysis and post-translational modifications, transcription regulation, signal transduction, and metabolism. The diversity of these roles indicates that XPC is involved in many more cellular processes than previously thought and provides a gateway for further understanding of the effects of xeroderma pigmentosum.

## 2. Results and Discussion

**Figure 1.**
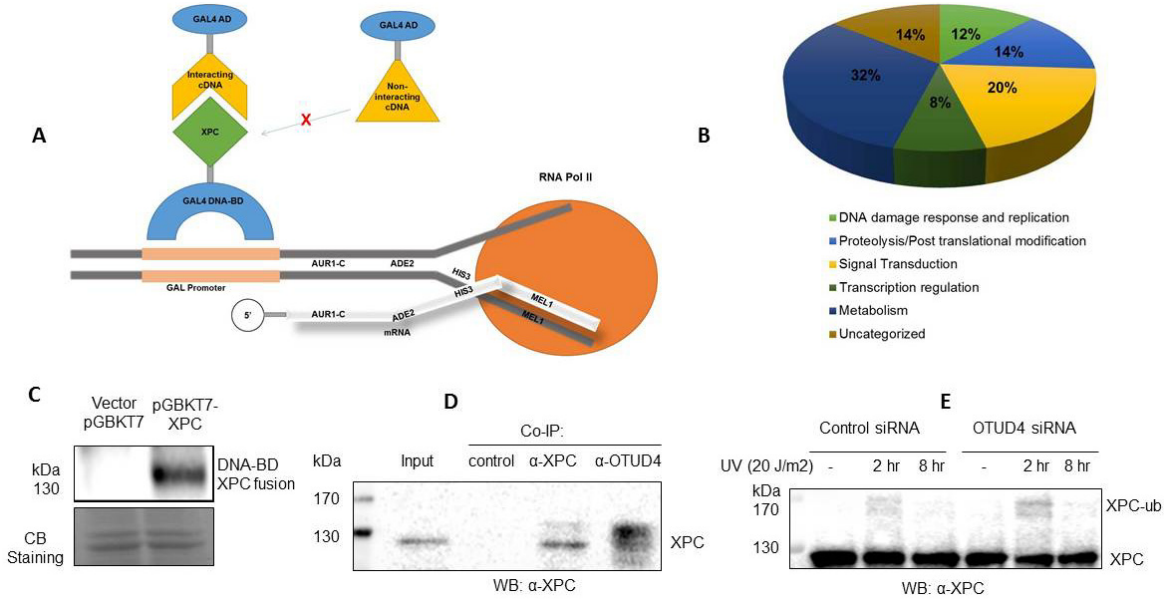
Schematic of the Yeast Two-Hybrid screening performed (**A**). The proximity of the DNA-BD and AD domains from the bait-prey interaction results in the transcription of four independent reporter genes (AUR1-C, ADE2, HIS3, and MEL1). See text for details; (**B**) Categorical organization of interactors of XPC identified by the yeast two-hybrid screening; (**C**) Confirmation of GAL4 DNA-BD-XPC fusion expression in yeast. Western blot was performed using an anti-XPC antibody (Cell Signaling). Coomassie blue (CB) staining was used as loading control; (**D**) Co-immunoprecipitation (Co-IP) to validate OTUD4-XPC interaction identified by the yeast two hybrid screening. HCT116 cell extracts were incubated with no antibody control, an XPC antibody, or an anti-OTUD4 antibody, respectively. Interacting proteins were collected by protein A/G beads and separated on a SDS-PAGE gel. Western blot was performed using a XPC antibody; and (**E**) Functional link between the OTUD4 deubiquitinase and XPC ubiquitination. Elevated levels of UV-induced XPC ubiquitination were detected in HCT116 cells with *OTUD4* knockdown.

In this study, using an improved yeast two-hybrid system (Figure 1A), we have identified 49 novel protein interactions with XPC. In order to further investigate the role of XPC within the cell, we have organized the functions into several categories: DNA repair and replication, proteolysis and post-translational modifications, transcription regulation, signal transduction, and metabolism (Figure 1B). While XPC has been known to play a major role in DNA damage and be modified by ubiquitin and ubiquitin-like factors, the other pathways indicated in this screening could represent novel functions of XPC and explain symptoms of xeroderma pigmentosum with as of yet unknown etiology.

The yeast two-hybrid system we used is the Matchmaker Gold Yeast Two-Hybrid System, an advanced, high performing system for investigating protein–protein interactions. As the proximity of the DNA-BD and AD domains from the bait-prey interaction results in the transcription of four independent reporter genes (*AUR1-C*, *ADE2*, *HIS3*, and *MEL1*) of a new yeast strain (Y2H Gold), the incidence of false positives is very low. The rate of false positives was also reduced by the use of the SMART-based Normalized Yeast Two-Hybrid cDNA Library which removes highly abundant transcripts to allow for the screening of low abundance and rare cDNAs. However, the use of the Universal Human version of this library, while allowing for a broad gene representation, decreased the chances of finding a single, specific interaction.

We first confirmed that a specific XPC antibody recognizes the DNA-BD-XPC fusion protein used in our Yeast Two-Hybrid system (Figure 1C). In addition, we have validated the OTUD4–XPC interaction in Human cells. We then focused on OTUD4, a novel deubiquitinase that we identified as an interactor with XPC. We found that XPC and OTUD4 interact directly in a co-immunoprecipitation experiment (Figure 1D). The co-IP indicated a potential affinity of OTUD4 for modified XPC, as demonstrated by antibody binding specifically to bands higher than the 130 kDa XPC band. Significantly, a knockdown of *OTUD4* in the human HCT116 cells results in an increase of ubiquitination of XPC or, perhaps, a decrease of deubiquitination of XPC in response to UV radiation (Figure 1E). The significant increase in XPC ubiquitination in response to a small knockdown of *OTUD4* (~30% knockdown of OTUD4) indicates a functional link between XPC and OTUD4. Our hypothesis is that the deubiquitinase OTUD4 is involved in XPC recycling by removing ubiquitin from ubiquitinated XPC. Experiments are ongoing to test this hypothesis.

The yeast two-hybrid screening identified 6 XPC-interacting proteins with functions of DNA repair and replication (Table 1). Interestingly, one of these proteins was XPC itself, indicating a potential dimerization of XPC in the course of NER. DNA damage-inducible transcript 3 (DDIT3) is upregulated in response to a variety of stressors and promotes apoptosis. *BRCA1*, a known tumor suppressor gene involved with the repair of double-stranded breaks (DSBs), induces the expression of DDIT3 in response to DNA damage induced by UV and doxorubicin in PC3 cells [26]. DDIT3 has since been implicated as a transcriptional regulator involved in the apoptotic response to DNA damage and endoplasmic reticulum (ER) stress [27,28]. For example, DDIT3 inhibits the expression of CCAAT/enhancer binding proteins (C/EBPs) as a dominant negative and activates the expression of AP-1 through interactions with Jun/Fos AP-1 complex proteins [29]. DDIT3 regulates apoptosis through regulation of PUMA and BLIM. DDIT3 expression is now used as a marker of ER stress. RPS3A is a ribosomal protein of the 40S subunit of the ribosome. In addition to its functions in ribosomal activity, RPS3A has been shown to upregulate NF-κB activity through chaperoning function [30,31] and play a role in preventing apoptosis in association with Bcl-2 and PARP [32–35]. Interestingly, RPS3A has been shown to interact with DDIT3. Considering the similar putative roles of RPS3A and DDIT3 in apoptosis in response to stress and interactions with Bcl-2, their interaction with each other, and their interactions with XPC, there is a possibility of a DDIT3-RPS3A-XPC regulated apoptotic pathway in response to DNA damage and other cell stressors. Support for a XPC-regulated UV damage inducible apoptotic pathway lies in the identification of XPC enhancing apoptosis in response to DNA damage [36]. PHC1 was identified in the yeast two-hybrid screening as interacting with XPC. PHC1 has previously been implicated in DNA damage repair; cells from patients with primary microcephaly (PM), a disease featuring a mutation in PHC1, showed impaired DNA damage repair in response to both ionized radiation and H_2_O_2_. Compared with control cells, IR of cells from PM patients resulted in a decrease in PHC1 association with chromatin, correlating with a decrease in ubiquitinated H2A and indicating a potential role of PHC1 in chromatin remodeling in response to DNA damage. The overexpression of wild-type PHC1 was sufficient to restore DNA damage repair including the ubiquitination of H2A to cells from PM patients [37]. Chromatin remodeling factors, including INO80 [38], Snf5 and BRG1 [39,40], components of the SWI/SNF chromatin remodeling complex, have been previously shown to aid in the recruitment of XPC to sites of DNA damage. PHC1 could function similarly in its putative role as a chromatin remodeling factor and recruit XPC to sites of DNA damage. Alternatively, XPC interacts with other chromatin remodeling factors to regulate downstream factors; XPC and hSNF5 interact to recruit and phosphorylate ATM [18] and it has been proposed that the XPC-BRG1 interaction functions to regulate chromatin relaxation and recruit XPG and PCNA [41]. The XPC-PHC1 interaction could function similarly in the recruitment of downstream NER factors.

The yeast two-hybrid screening identified 7 XPC-interacting proteins with functions of post-translational modification/proteolysis (Table 2). OTUD4, a putative deubiquitinase, was identified. Many cellular proteins are stabilized posttranslationally by deubiquitination, which is carried out by a class of enzymes called deubiquitinases (DUBs). DUBs remove the polyubiquitin chains from their substrates and thereby increase their cellular pool. XPC is ubiquitinated after UV, but ubiquitinated XPC does not appear to be directed for degradation [6]. We speculate that deubiquitination of XPC by OTUD4 could be responsible for the removal of XPC from damage sites, possibly leading to XPC recycling. Indeed, our data presented in Figure 1 support that there is a functional link between OTUD4 and XPC in human cells. We also identified PSMA4 as interacting with XPC. Although current data indicate that XPC is not sent to the proteasome for degradation following ubiquitination, it has been postulated that XPC interacts directly with the proteasome to signal its degradation. Further, it has been shown that the 19S subunit of the proteasome positively modulates NER while inhibition of the 20S subunit, of which PSMA4 is a part, reduces the recruitment of XPC to damaged sites. This interaction could be responsible for signaling XPC degradation or for recruiting XPC to damaged sites. Ubiquitin-like modifier activating enzyme 3 (UBA3) was identified in the yeast two-hybrid screening. UBA3 binds with NEDD8 activating enzyme E1 subunit 1 (NAE1) to form a heterodimer that serves as the sole E1 enzyme of neddylation and is implicated in cell cycle progression. Since NEDD8 accumulates in response to UV radiation, we speculate that XPC, in its interaction with UBA3, initiates a neddylation cascade in response to its recognition of DNA damage. It has previously been shown that neddylation of histone H4 occurs in response to DNA damage by ionized radiation [43]; therefore, H4 could also be the target of neddylation in NER.

The yeast two-hybrid screening identified 10 XPC-interacting proteins with functions in signal transduction (Table 3). The MAPK signaling pathway involves a family of serine threonine kinases activated in response to different cell stressors. Several proteins within the MAPK signaling pathway have been identified as playing a role in GG-NER. For example, p300, a co-transcriptional factor, is phosphorylated by p38 and AKT kinases of the MAPK family and then acetylates histones to allow DNA damage recognition factors such as XPC to access damaged DNA [50]. Regulation of XPC itself has also been attributed to the MAPK family: p38 MAPK is required for the recruitment of XPC and TFIIH to damaged DNA sites [51]. The identification of mitogen-activated protein kinase kinase kinase 5 (MAP3K5), a kinase within the MAPK family whose roles include activation of the p38 pathway, as interacting with XPC further supports the idea of the recruitment of XPC to DNA damage sites through MAPK signaling. Further, PTEN, a regulator of AKT signaling within the p38 MAPK pathway, has been shown to positively regulate the transcription of XPC [52]. Protein tyrosine phosphatase type IVA, member 2 (PTP4A2), also known as PRL2, was recently shown to down regulate PTEN, promoting AKT signaling. It can be extrapolated that this downregulation of PTEN decreases the transcription of XPC and, therefore, the recognition of lesions within GG-NER. Identified by our Y2H screening as interacting with XPC, PTP4A2 could potentially be a negative regulator of XPC and the DNA damage response. Another signaling pathway associated with the regulation of GG-NER is the PKA pathway. PKA, involved in mitotic regulation and chromatin remodeling, has been implicated in DNA damage repair through its phosphorylation of p19INK4d which promotes the DNA damage response after a cell is subject to UV radiation, β-amyloid peptides, and cisplatin; its phosphorylation of Cdc20, a factor involved in DNA damage checkpoints; and its potential phosphorylation of Pol δ, a DNA polymerase involved in DNA repair and replication. PKA comprises two regulatory subunits and two catalytic subunits, all of which have been identified as existing in different isoforms. The yeast two-hybrid screening identified two isoforms of the catalytic subunit of PKA—PRKACB and PRKACA—as potential interactors of XPC. PRKACA, in particular, has been suggested to play a role in the DNA damage response pathway through its translocation-causing phosphorylation of S100A11, a stress response protein associated with p21. PRKACB or PRKACA could stimulate GG-NER through chromatin remodeling or translocation of XPC into the nucleus.

In addition to XPC-interacting proteins with functions of DNA repair and replication, proteolysis and post-translational modifications, and signal transduction, the yeast two-hybrid screening identified 4 XPC-interacting proteins with functions of transcription regulation (Table 4), 15 XPC-interacting proteins with functions of metabolism (Table 5), and 7 XPC-interacting proteins with uncategorized functions (Table 6).

## 3. Experimental Section

### 3.1. Yeast Two-Hybrid Screen

A Matchmaker-Gold yeast two-hybrid screening was performed according to manufacturer’s instruction. Briefly, the full-length of the coding region of XPC was inserted in frame into the multiple cloning sites of the DNA-BD vector, pGBKT7 (Clontech), to generate the bait plasmid pGBKT7-XPC, which was subsequently confirmed by sequencing. The pGBKT7-XPC was transformed into the bait strain Y2HGold and XPC expression was confirmed by Western bloting using a specific human XPC antibody (Figure 1). XPC bait strain Y2HGold was mated with the pre-transformed Y187/pACT2 normalized universal human Mate & Plate cDNA library according to the Clontech protocol. Diploid yeast cells were plated on a nutrient deficiency medium SD plate without Trp and Leu (DDO) and analyzed for their ability to grow in the presence of highly toxic drug Aureobasidin A (125 ng/mL, Clontech) and regulate α-galactosidase expression, which hydrolyzes 5-Bromo-4-chloro-3-indolyl-a-d-galactopyranoside (X-α-gal, 40 μg/mL; Clontech) to produce a blue-end product. The selected colonies were restreaked on SD plate without Trp, Leu, His and Ade (QDO) containing Aureobasidin A and X-α-gal for further selection.

### 3.2. Sequencing and Sequencing Data Analysis

Plasmid DNA from yeast was isolated and transformed into *Escherichia coli* DH5*a* for propagation. Plasmid DNA from *E. coli* was then sequenced (http://www.genewiz.com/). Nucleotide and deduced protein sequences were identified using BLAST [72] and EMBL-EBI [73].

## 4. Conclusions

Our high-throughput Yeast Two Hybrid screening identified 49 novel proteins that interact with XPC with roles in DNA repair and replication, proteolysis and post-translational modifications, transcription regulation, signal transduction, and metabolism, helping to elucidate the interactome of XPC. We validated the XPC-OTUD4 interaction by co-IP and demonstrated that OTUD4 knockdown in human cells indeed affects the levels of ubiquitinated XPC, supporting a hypothesis that the OTUD4 deubiquitinase is involved in XPC recycling by removing the ubiquitin moiety. It must be noted that several well-known interactors of XPC were not identified in the Y2H screening, namely RAD23B and CETN2. This lack of identification does not detract from the novel interactions identified by the screening, but rather indicates the extent to which an interactome can be defined by a single yeast two-hybrid screening or library variation. Further Y2H screenings involving XPC, RAD23B, or CETN2 in various libraries have the potential to demonstrate the interactions previously reported. Previous Y2H screenings with interactions further supported by other methods indicate that the accuracy of these screenings is over 60%. Therefore, we expect that at least half of the protein interactions identified in this study may provide a meaningful gateway for future exploration. Understanding the interactome of XPC is crucial in determining the mechanism of DNA damage recognition in the context of chromatin and the pathogenesis of xeroderma pigmentosum. This study is the jumping off point for numerous investigations into the role of XPC in the development of XP, GG-NER, and as yet undiscovered roles within the cell.

## Figures and Tables

**Table 1.** DNA damage response and replication (6 proteins).

Gene	Full name	Accession No.	Brief description
*XPC*	xeroderma pigmentosum, complementation group C	NP_001139241.1	XPC forms a complex with RAD23B and CETN2 [1] and serves to recognize DNA damage (specifically bulky lesions caused by factors such as UV and cisplatin) and recruit TFIIH to the damaged sites [14,15], initiating global genomic nucleotide excision repair.
*DDIT3*	DNA damage-inducible transcript 3	NP_001181982.1	DDIT3 has since been implicated as a transcriptional regulator involved in the apoptotic response to DNA damage and endoplasmic reticulum (ER) stress [27,28].
*MNDA*	myeloid cell nuclear differentiation antigen	NP_002423.1	MNDA is involved in the immune response and has putative roles in transcription regulation and interferon response.
*RRM1*	ribonucleotide reductase M1	NP_001024.1	RRM1 is the large subunit of ribonucleoside-diphosphate reductase, the enzyme responsible for synthesis of deoxyribonucleotides in preparation for DNA synthesis in S phase.
*PHC1*	polyhomeotic homolog 1	NP_004417.2	PHC1, the human homolog of *Drosophila* Ph-p, is part of a Polycomb repressive complex, hPRC-H, a Polycomb group (PcG) complex involved in expression regulation through transcription repression by blocking chromatin remodeling by SWI/SNF [42]. Recently, PHC1 has been implicated as playing a role in the DNA damage response pathway, potentially through cell cycle regulation, chromatin remodeling, and the ubiquitination of H2A [37].
*RPS3A*	ribosomal protein S3A	NP_000997.1	RPS3A is a component of the 40S subunit of the ribosome.

**Table 2.** Proteolysis/Post translational modification (7 proteins).

Gene	Full name	Accession No.	Brief description
*PREP*	prolyl endopeptidase	NP_002717.3	PREP is a cytoplasmic endoprotease which cleaves proteins through hydrolysis at the *C*-terminal side of proline. PREP has been implicated as playing a role in numerous disorders such as cancer and hypertension though a majority is associated with the nervous system with roles in neurodegenerative disorders and neuronal differentiation and maturation. Further, PREP appears to have many roles within the cell including cell death, protein secretion, and cell differentiation and proliferation. These roles and functions were reviewed by [44] and more support has come out recently for PREP’s roles in the cell cycle [45], cell death [46], and cell signaling [47] in neuroblastoma cell lines.
*DLD*	dihydrolipoamide dehydrogenase	NP_000099.2	DLD is the E3 component of the pyruvate dehydrogenase and α-ketoglutarate dehydrogenase complexes with numerous oxidoreductase functions including catalyzing the oxidative regeneration of E2 lipoic acid cofactors, catalyzing the oxidation of NADH to NAD^+^, and proteolysis [48].
*UBA3*	ubiquitin-like modifier activating enzyme 3	NP_003959.3	In complex with NAE1, UBA3 serves as the E3 ligase during neddylation.
*PSMA4*	proteasome subunit, alpha type, 4	NP_001096137.1	PSMA4, a member of the peptidase T1A family, encodes a subunit of the 20S proteasome.
*OTUD4*	OTU domain containing 4	NP_001096123.1	OTUD4 is a putative deubiquitinase.
*UBLCP1*	ubiquitin-like domain containing CTD phosphatase 1	NP_659486.2	UBLCP1 dephosphorylates the 26S nuclear proteasome, preventing its maturation and function.
*ACTR6*	ARP6 actin-related protein 6 homolog	NP_071941.1	The yeast homolog of ACTR6, Arp6, acts in complex with Swc3, Swc2, Swc6 as part of the SWR1 complex to remodel chromosomes. This module of proteins replaces variant histone H2A.Z, placed in the chromosome to control function, with H2A. Arp6 has been shown to be requisite for association of Swc2 and for nucleosome binding. Though the role of ACTR6 has not been analyzed in humans, the conservation of the SR1 complex across several species (yeast, Drosophila with a role in heterochromatin formation, and human) indicates a role of chromatin in remodeling in humans by ACTR6 [49].

**Table 3.** Signal transduction (10 proteins).

Gene	Full name	Accession No.	Brief description
*PRKACA*	PRKACA protein kinase, cAMP-dependent, catalytic, alpha	NP_002721.1	PRKACA is an isoform of the catalytic subunit of PKA, a cAMP-dependent protein kinase of the Ser/Thr protein kinase family and functions in numerous biological processes throughout the cell including tight junction formation, response to glucose, and regulation of the proteasome. PKA is also activated with the c-Myc-induced transcription of PRKACB, an alternative isoform of the catalytic subunit.
*MAP3K5*	mitogen-activated protein kinase kinase kinase 5	NP_005914.1	MAP3K5, a member of the MAPK family of serine threonine kinase, functions in the MKK/JNK signal transduction cascade and the p38 MAPK signal transduction cascade and has roles in apoptosis and the immune response.
*TSPAN6*	tetraspanin 6	NP_001265669.1	TSPAN6 was originally identified in large scale screening as activating the NF-κB pathway [53] and characterized as part of the transmembrane 4 superfamily with high expression in in the liver, pancreas, kidney, ovary, and ependymoma 383. More recently, TSPAN6 has been implicated in the negative regulation of RLR signaling (specifically the activation of NF-κB and IFN-β promoters) through its ubiquitin-dependent association with MAVS [54].
*EMP2*	epithelial membrane protein 2	NP_001415.1	Part of the transmembrane 4 superfamily, EMP2 has been shown to play a role in blastocyst implantation [55], the regulation of cell surface proteins such as caveolin-1 [56] and αvβ3 integrin [57], the carcinogenesis of endometrial tumors [58,59], and, potentially, chlamydia [60]. Further, progesterone up-regulates EMP2 expression [61] and has been postulated a target for the treatment of ovarian cancer [62].
*SRGN*	serglycin	NP_002718.2	SRGN is a granule proteoglycan with several roles in the immune response such as granule storage and localization within neutrophils. SRGN also is involved with apoptosis and the regulation of TNF-α secretion.
*RAB1A*	member RAS oncogene family	NP_004152.1	RAB1A is a member of the Ras superfamily of GTPases and functions in protein and vesicle transport in the ER and Golgi body.
*TJP1*	tight junction protein 1	NP_003248.3	TJP1 is a protein associated with cell–cell junctions that is involved in signal transduction, tight junction assembly and stability, and cell migration.
*SPCS1*	signal peptidase complex subunit 1	NP_054760.3	The function of SPCS1, a subunit of the mammalian signal peptidase complex that spans the ER membrane twice with both termini residing in the cytosol [63], mainly unknown though its yeast homolog, Spc1p, contributes non-essentially to signal peptidase efficiency [64].
*PTP4A2*	protein tyrosine phosphatase type IVA, member 2	NP_001182029.1	PTP4A2, a member of the protein tyrosine phosphatase (PTP) family, is a phosphatase of regenerating liver with oncogenic properties that downregulates PTEN and is involved in cell cycle progression.
*TRGV4*	T cell receptor gamma variable 4		TRGV4 is a T cell receptor involved in the immune response.

**Table 4.** Transcription regulation (4 proteins).

Gene	Full name	Accession No.	Brief description
*BBX*	bobby sox homolog	NP_001136040.1	Mammalian BBX was shown in yeast to activate Cdc10-dependent transcription at the G1/S transition of the cell cycle.
*RALGAPA1*	Ral GTPase activating protein, alpha subunit 1	NP_055805.1	RALGAPA1 is the catalytic subunit of RALGAP1, the GTPase-activating protein which drastically increases the GTP hydrolysis rate of RalA [65].
*BAZ2B*	bromodomain adjacent to zinc finger domain, 2B	NP_038478.2	BAZ2B, a member of the novel bromodomain family, recognizes histone and is involved in chromatin-dependent regulation of transcription.
*DHX32*	DEAH (Asp-Glu-Ala-His) box polypeptide 32	NP_060650.2	DEAH, a member of the DEAD box proteins, is a RNA helicase which localizes to the nucleus and mitochondria [66]. DHX32 has been implicated in lymphoid maturation [67] and the immune response [68].

**Table 5.** Metabolism (15 proteins).

Gene	Full name	Accession No.	Brief description
*PLBD1*	phospholipase B domain containing 1	NP_079105.4	PLBD1 is a phospholipase thought to play a role in the immune response due to the identification of a precursor in neutrophils [69].
*PPA1*	pyrophosphatase (inorganic) 1	NP_066952.1	PPA1 is a member of the inorganic pyrophosphatase (PPase) family with a potential cytoplasmic location.
*FMO3*	flavin containing monooxygenase 3	NP_001002294.1	FMO3 catalyzes the oxidative metabolism of xenobiotics and the *S*-oxidation of sulfide compounds.
*PDXDC1*	pyridoxal-dependent decarboxylase domain containing 1	NP_055842.2	PDXDC1 maps to the glycerophospholipid and sphingolipid metabolism pathways and is strongly associated with the ratios of lysophosphatidylcholines (20:3), phosphatidylethanolamines, and phosphatidylcholines [70].
*ASS1*	argininosuccinate synthase 1	NP_000041.2	ASS1 functions in the arginine biosynthetic pathway.
*FUNDC1*	FUN14 domain containing 1	NP_776155.1	FUNDC1, a member of the FUN14 superfamily, functions in hypoxia-induced mitophagy.
*CISD2*	CDGSH iron sulfur domain 2	NP_001008389.1	CISD2 is a zinc finger protein in the ER that is involved in autophagy.
*RPP30*	ribonuclease P/MRP 30kDa subunit	NP_001098016.1	RPP30 is a component of ribonuclease P, an enzyme that catalyzes the maturation of tRNA molecules.
*TRMT13*	tRNA methyltransferase 13 homolog	NP_061956.2	TRMT13 is a putative tRNA methylase.
*SPAG16*	SPAG16 sperm associated antigen 16	NP_001020607.1	SPAG16 is localized to the sperm and is involved in sperm flagellum function.
*CEP170*	centrosomal protein 170kDa	NP_001035863.1	CEP170 is a component of the centrosome and is involved in the maintenance of microtubule organization and cell morphology.
*EMCN*	endomucin	NP_001153166.1	EMCN, a sialoglycoprotein, interferes with focal adhesion complexes and cell-extracellular matrix interactions.
*MRPL50*	mitochondrial ribosomal protein L50	NP_061924.1	MRPL50, part of the L47P ribosomal protein family, is a potential component of the mitochondrial ribosome.
*TRAPPC4*	trafficking protein particle complex 4	NP_057230.1	TRAPPC4 has a putative role in vesicle transport.
*MFSD1*	major facilitator superfamily domain containing 1	NP_001161375.1	MFSD1 has been bioinformatically classified as a part of the superfamily of solute carriers (SLCs) [71].

**Table 6.** Unclassified (7 proteins).

Gene	Full name	Accession No.	Brief description
*OLA1*	Obg-like ATPase 1	NP_001011708.1	DNA damage-regulated overexpressed in cancer 45 protein. Possible role in cell proliferation and survival.
*ZMAT1*	zinc finger, matrintype 1	NP_001011657.2	A protein containing Cys2–His2 (C2H2)-type zinc fingers, which are similar to those found in the nuclear matrix protein matrin 3.
*MAGEB10*	melanoma antigen family B, 10	NP_872312.2	A member of the B subfamily of the melanoma associated antigen protein family. The encoded protein is specifically expressed in testis and tumor cells.
*HSDL2*	hydroxysteroid dehydrogenase like 2	NP_001182751.1	Short Chain Dehydrogenase/Reductase Family.
*DSEL*	dermatan sulfate epimerase-like	NP_115536.1	Function unknown.
*TMEM248*	transmembrane protein 248	NP_060464.1	Function unknown.
*PRRC2C*	proline-rich coiled-coil 2C	NP_055987.2	Function unknown.

## References

[1] Araki M., Masutani C., Takemura M., Uchida A., Sugasawa K., Kondoh J., Ohkuma Y., Hanaoka F. (2001). Centrosome protein centrin 2/caltractin 1 is part of the xeroderma pigmentosum group C complex that initiates global genome nucleotide excision repair. J. Biol. Chem.

[2] Bootsma D., Hoeijmakers J.H. (1991). The genetic basis of xeroderma pigmentosum. Ann. Genet.

[3] Khan S.G., Metin A., Gozukara E., Inui H., Shahlavi T., Muniz-Medina V., Baker C.C., Ueda T., Aiken J.R., Schneider T.D. (2004). Two essential splice lariat branchpoint sequences in one intron in a xeroderma pigmentosum DNA repair gene: Mutations result in reduced XPC mRNA levels that correlate with cancer risk. Hum. Mol. Genet.

[4] Hananian J., Cleaver J.E. (1980). Xeroderma pigmentosum exhibiting neurological disorders and systemic lupus erythematosus. Clin. Genet.

[5] Khan S.G., Levy H.L., Legerski R., Quackenbush E., Reardon J.T., Emmert S., Sancar A., Li L., Schneider T.D., Cleaver J.E. (1998). Xeroderma pigmentosum group C splice mutation associated with autism and hypoglycinemia. J. Invest. Dermatol.

[6] Sugasawa K. (2006). UV-induced ubiquitylation of XPC complex, the UV-DDB-ubiquitin ligase complex, and DNA repair. J. Mol. Histol.

[7] Poulsen S.L., Hansen R.K., Wagner S.A., van Cuijk L., van Belle G.J., Streicher W., Wikstrom M., Choudhary C., Houtsmuller A.B., Marteijn J.A. (2013). RNF111/Arkadia is a SUMO-targeted ubiquitin ligase that facilitates the DNA damage response. J. Cell Biol.

[8] Batty D., Rapic’-Otrin V., Levine A.S., Wood R.D. (2000). Stable binding of human XPC complex to irradiated DNA confers strong discrimination for damaged sites. J. Mol. Biol.

[9] Krasikova Y.S., Rechkunova N.I., Maltseva E.A., Craescu C.T., Petruseva I.O., Lavrik O.I. (2012). Influence of centrin 2 on the interaction of nucleotide excision repair factors with damaged DNA. Biochemistry.

[10] Bunick C.G., Miller M.R., Fuller B.E., Fanning E., Chazin W.J. (2006). Biochemical and structural domain analysis of xeroderma pigmentosum complementation group C protein. Biochemistry.

[11] Masutani C., Araki M., Sugasawa K., van der Spek P.J., Yamada A., Uchida A., Maekawa T., Bootsma D., Hoeijmakers J.H., Hanaoka F. (1997). Identification and characterization of XPC-binding domain of hHR23B. Mol. Cell. Biol.

[12] Li L., Lu X., Peterson C., Legerski R. (1997). XPC interacts with both HHR23B and HHR23A *in vivo*. Mutat. Res..

[13] Sugasawa K., Ng J.M., Masutani C., Maekawa T., Uchida A., van der Spek P.J., Eker A.P., Rademakers S., Visser C., Aboussekhra A. (1997). Two human homologs of Rad23 are functionally interchangeable in complex formation and stimulation of XPC repair activity. Mol. Cell. Biol.

[14] Araujo S.J., Nigg E.A., Wood R.D. (2001). Strong functional interactions of TFIIH with XPC and XPG in human DNA nucleotide excision repair, without a preassembled repairosome. Mol. Cell. Biol.

[15] Yokoi M. (2000). The xeroderma pigmentosum Group C protein complex XPC-HR23B plays an important role in the recruitment of transcription factor IIH to damaged DNA. J. Biol. Chem.

[16] Thoma B.S., Wakasugi M., Christensen J., Reddy M.C., Vasquez K.M. (2005). Human XPC-hHR23B interacts with XPA-RPA in the recognition of triplex-directed psoralen DNA interstrand crosslinks. Nucl. Acid. Res.

[17] Lange S.S., Reddy M.C., Vasquez K.M. (2009). Human HMGB1 directly facilitates interactions between nucleotide excision repair proteins on triplex-directed psoralen interstrand crosslinks. DNA Repair.

[18] Ray A., Mir S.N., Wani G., Zhao Q., Battu A., Zhu Q., Wang Q.E., Wani A.A. (2009). Human SNF5/INI1, a component of the human SWI/SNF chromatin remodeling complex, promotes nucleotide excision repair by influencing ATM recruitment and downstream H2AX phosphorylation. Mol. Cell. Biol.

[19] Zhu Q., Wani G., Arab H.H., El-Mahdy M.A., Ray A., Wani A.A. (2009). Chromatin restoration following nucleotide excision repair involves the incorporation of ubiquitinated H2A at damaged genomic sites. DNA Repair.

[20] Shimizu Y., Iwai S., Hanaoka F., Sugasawa K. (2003). Xeroderma pigmentosum group C protein interacts physically and functionally with thymine DNA glycosylase. EMBO J.

[21] Fong Y.W., Inouye C., Yamaguchi T., Cattoglio C., Grubisic I., Tjian R. (2011). A DNA repair complex functions as an Oct4/Sox2 coactivator in embryonic stem cells. Cell.

[22] Havugimana P.C., Hart G.T., Nepusz T., Yang H., Turinsky A.L., Li Z., Wang P.I., Boutz D.R., Fong V., Phanse S. (2012). A census of human soluble protein complexes. Cell.

[23] Lehner B., Sanderson C.M. (2004). A protein interaction framework for human mRNA degradation. Genome Res.

[24] Colland F., Jacq X., Trouplin V., Mougin C., Groizeleau C., Hamburger A., Meil A., Wojcik J., Legrain P., Gauthier J.M. (2004). Functional proteomics mapping of a human signaling pathway. Genome Res.

[25] Montes de Oca R., Shoemaker C.J., Gucek M., Cole R.N., Wilson K.L. (2009). Barrier-to-autointegration factor proteome reveals chromatin-regulatory partners. PLoS One.

[26] De Luca P., Vazquez E.S., Moiola C.P., Zalazar F., Cotignola J., Gueron G., Gardner K., de Siervi A. (2011). BRCA1 loss induces GADD153-mediated doxorubicin resistance in prostate cancer. Mol. Cancer Res.

[27] Su N., Kilberg M.S. (2008). C/EBP homology protein (CHOP) interacts with activating transcription factor 4 (ATF4) and negatively regulates the stress-dependent induction of the asparagine synthetase gene. J. Biol. Chem.

[28] Yamaguchi H., Wang H.G. (2004). CHOP is involved in endoplasmic reticulum stress-induced apoptosis by enhancing DR5 expression in human carcinoma cells. J. Biol. Chem.

[29] Horndasch M., Lienkamp S., Springer E., Schmitt A., Pavenstadt H., Walz G., Gloy J. (2006). The C/EBP homologous protein CHOP (GADD153) is an inhibitor of Wnt/TCF signals. Oncogene.

[30] Lim K.H., Kim K.H., Choi S.I., Park E.S., Park S.H., Ryu K., Park Y.K., Kwon S.Y., Yang S.I., Lee H.C. (2011). RPS3a over-expressed in HBV-associated hepatocellular carcinoma enhances the HBx-induced NF-kappaB signaling via its novel chaperoning function. PLoS One.

[31] Wan F., Lenardo M.J. (2010). The nuclear signaling of NF-kappaB: current knowledge, new insights, and future perspectives. Cell Res.

[32] Naora H. (1999). Involvement of ribosomal proteins in regulating cell growth and apoptosis: Translational modulation or recruitment for extraribosomal activity?. Immun. Cell Biol.

[33] Russell L., Naora H., Naora H. (2000). Down-regulated RPS3a/nbl expression during retinoid-induced differentiation of HL-60 cells: A close association with diminished susceptibility to actinomycin d-stimulated apoptosis. Cell Struct. Funct.

[34] Naora H., Takai I., Adachi M., Naora H. (1998). Altered cellular responses by varying expression of a ribosomal protein gene: Sequential coordination of enhancement and suppression of ribosomal protein S3a gene expression induces apoptosis. J. Cell. Biol.

[35] Song D., Sakamoto S., Taniguchi T. (2002). Inhibition of poly (ADP-ribose) polymerase activity by Bcl-2 in association with the ribosomal protein S3a. Biochemistry.

[36] Wang Q.E., Han C., Zhang B., Sabapathy K., Wani A.A. (2012). Nucleotide excision repair factor XPC enhances DNA damage-induced apoptosis by downregulating the antiapoptotic short isoform of caspase-2. Cancer Res.

[37] Awad S., Al-Dosari M.S., Al-Yacoub N., Colak D., Salih M.A., Alkuraya F.S., Poizat C. (2013). Mutation in PHC1 implicates chromatin remodeling in primary microcephaly pathogenesis. Hum. Mol. Genet.

[38] Jiang Y., Wang X., Bao S., Guo R., Johnson D.G., Shen X., Li L. (2010). INO80 chromatin remodeling complex promotes the removal of UV lesions by the nucleotide excision repair pathway. Proc. Natl. Acad. Sci. USA.

[39] Zhang L., Zhang Q., Jones K., Patel M., Gong F. (2009). The chromatin remodeling factor BRG1 stimulates nucleotide excision repair by facilitating recruitment of XPC to sites of DNA damage. Cell Cycle.

[40] Zhang L., Chen H., Gong M., Gong F. (2013). The chromatin remodeling protein BRG1 modulates BRCA1 response to UV irradiation by regulating ATR/ATM activation. Front. Oncol.

[41] Zhao Q., Wang Q.E., Ray A., Wani G., Han C., Milum K., Wani A.A. (2009). Modulation of nucleotide excision repair by mammalian SWI/SNF chromatin-remodeling complex. J. Biol. Chem.

[42] Levine S.S., Weiss A., Erdjument-Bromage H., Shao Z., Tempst P., Kingston R.E. (2002). The core of the polycomb repressive complex is compositionally and functionally conserved in flies and humans. Mol. Cell. Biol.

[43] Ma T., Chen Y., Zhang F., Yang C.Y., Wang S., Yu X. (2013). RNF111-dependent neddylation activates DNA damage-induced ubiquitination. Mol. Cell.

[44] Brandt I., Scharpe S., Lambeir A.M. (2007). Suggested functions for prolyl oligopeptidase: A puzzling paradox. Clin. Chim. Acta.

[45] Sakaguchi M., Matsuda T., Matsumura E., Yoshimoto T., Takaoka M. (2011). Prolyl oligopeptidase participates in cell cycle progression in a human neuroblastoma cell line. Biochem. Biophys. Res. Commun.

[46] Matsuda T., Sakaguchi M., Tanaka S., Yoshimoto T., Takaoka M. (2013). Prolyl oligopeptidase is a glyceraldehyde-3-phosphate dehydrogenase-binding protein that regulates genotoxic stress-induced cell death. Int. J. Biochem. Cell Biol.

[47] Moreno-Baylach M.J., Puttonen K.A., Tenorio-Laranga J., Venalainen J.I., Storvik M., Forsberg M.M., Garcia-Horsman J.A. (2011). Prolyl endopeptidase is involved in cellular signalling in human neuroblastoma SH-SY5Y cells. Neurosignals.

[48] Babady N.E., Pang Y.P., Elpeleg O., Isaya G. (2007). Cryptic proteolytic activity of dihydrolipoamide dehydrogenase. Proc. Natl. Acad. Sci. USA.

[49] Yoshida T., Shimada K., Oma Y., Kalck V., Akimura K., Taddei A., Iwahashi H., Kugou K., Ohta K., Gasser S.M. (2010). Actin-related protein Arp6 influences H2A.Z-dependent and independent gene expression and links ribosomal protein genes to nuclear pores. PLoS Genet.

[50] Ohoka N., Hattori T., Kitagawa M., Onozaki K., Hayashi H. (2007). Critical and functional regulation of CHOP (C/EBP homologous protein) through the *N*-terminal portion. J. Biol. Chem.

[51] Zhao Q., Barakat B.M., Qin S., Ray A., El-Mahdy M.A., Wani G., Arafa el S., Mir S.N., Wang Q.E., Wani A.A. (2008). The p38 mitogen-activated protein kinase augments nucleotide excision repair by mediating DDB2 degradation and chromatin relaxation. J. Biol. Chem.

[52] Ming M., Feng L., Shea C.R., Soltani K., Zhao B., Han W., Smart R.C., Trempus C.S., He Y.Y. (2011). PTEN positively regulates UVB-induced DNA damage repair. Cancer Res.

[53] Matsuda A., Suzuki Y., Honda G., Muramatsu S., Matsuzaki O., Nagano Y., Doi T., Shimotohno K., Harada T., Nishida E. (2003). Large-scale identification and characterization of human genes that activate NF-kappaB and MAPK signaling pathways. Oncogene.

[54] Wang Y., Tong X., Omoregie E.S., Liu W., Meng S., Ye X. (2012). Tetraspanin 6 (TSPAN6) negatively regulates retinoic acid-inducible gene I-like receptor-mediated immune signaling in a ubiquitination-dependent manner. J. Biol. Chem.

[55] Wadehra M., Dayal M., Mainigi M., Ord T., Iyer R., Braun J., Williams C.J. (2006). Knockdown of the tetraspan protein epithelial membrane protein-2 inhibits implantation in the mouse. Dev. Biol.

[56] Forbes A., Wadehra M., Mareninov S., Morales S., Shimazaki K., Gordon L.K., Braun J. (2007). The tetraspan protein EMP2 regulates expression of caveolin-1. J. Biol. Chem.

[57] Wadehra M., Forbes A., Pushkarna N., Goodglick L., Gordon L.K., Williams C.J., Braun J. (2005). Epithelial membrane protein-2 regulates surface expression of alphavbeta3 integrin in the endometrium. Dev. Biol.

[58] Shimazaki K., Lepin E.J., Wei B., Nagy A.K., Coulam C.P., Mareninov S., Fu M., Wu A.M., Marks J.D., Braun J. (2008). Diabodies targeting epithelial membrane protein 2 reduce tumorigenicity of human endometrial cancer cell lines. Clin. Cancer Res.

[59] Fu M., Rao R., Sudhakar D., Hogue C.P., Rutta Z., Morales S., Gordon L.K., Braun J., Goodglick L., Wadehra M. (2011). Epithelial membrane protein-2 promotes endometrial tumor formation through activation of FAK and Src. PLoS One.

[60] Shimazaki K., Wadehra M., Forbes A., Chan A.M., Goodglick L., Kelly K.A., Braun J., Gordon L.K. (2007). Epithelial membrane protein 2 modulates infectivity of Chlamydia muridarum (MoPn). Microbes Infect.

[61] Wadehra M., Mainigi M., Morales S.A., Rao R.G., Gordon L.K., Williams C.J., Braun J. (2008). Steroid hormone regulation of EMP2 expression and localization in the endometrium. Reprod. Biol. Endocrinol.

[62] Fu M., Maresh E.L., Soslow R.A., Alavi M., Mah V., Zhou Q., Iasonos A., Goodglick L., Gordon L.K., Braun J. (2010). Epithelial membrane protein-2 is a novel therapeutic target in ovarian cancer. Clin. Cancer Res.

[63] Hartmann E. (1996). Membrane Topology of the 12- and the 25-kDa subunits of the mammalian signal peptidase complex. J. Biol. Chem.

[64] Panzner S. (1996). The Homologue of mammalian SPC12 is important for efficient signal peptidase activity in *Saccharomyces cerevisiae*. J. Biol. Chem.

[65] Shirakawa R., Fukai S., Kawato M., Higashi T., Kondo H., Ikeda T., Nakayama E., Okawa K., Nureki O., Kimura T. (2009). Tuberous sclerosis tumor suppressor complex-like complexes act as GTPase-activating proteins for Ral GTPases. J. Biol. Chem.

[66] Alli Z., Ackerley C., Chen Y., Al-Saud B., Abdelhaleem M. (2006). Nuclear and mitochondrial localization of the putative RNA helicase DHX32. Exp. Mol. Pathol.

[67] Alli Z., Ho M., Abdelhaleem M. (2005). Expression of DHX32 in lymphoid tissues. Exp. Mol. Pathol.

[68] Alli Z., Nam E.H., Beimnet K., Abdelhaleem M. (2005). The activation-induced expression of DHX32 in Jurkat T cells is specific and involves calcium and nuclear factor of activated T cells. Cell. Immun.

[69] Xu S., Zhao L., Larsson A., Venge P. (2009). The identification of a phospholipase B precursor in human neutrophils. FEBS J.

[70] Demirkan A., van Duijn C.M., Ugocsai P., Isaacs A., Pramstaller P.P., Liebisch G., Wilson J.F., Johansson A., Rudan I., Aulchenko Y.S. (2012). Genome-wide association study identifies novel loci associated with circulating phospho- and sphingolipid concentrations. PLoS Genet.

[71] Sreedharan S., Stephansson O., Schioth H.B., Fredriksson R. (2011). Long evolutionary conservation and considerable tissue specificity of several atypical solute carrier transporters. Gene.

[72] BLAST Assembled RefSeq Genomes.

[73] A Fast Browser for Gene Ontology Terms and Annotations.

